# Genome-Wide Identification and Expression Analysis of PP2C Gene Family in Eelgrass

**DOI:** 10.3390/genes16060657

**Published:** 2025-05-29

**Authors:** Chang Liu, Xu Dong, Dazuo Yang, Qingchao Ge, Jiaxin Dai, Zhi Ma, Rongna Wang, Huan Zhao

**Affiliations:** 1 Key Laboratory of Marine Bio-Resources Restoration and Habitat Reparation in Liaoning Province, Dalian Ocean University, Dalian 116023, China; liuchang_bio@foxmail.com (C.L.);; 2 Department of Nursing, Zibo Vocational Institute, Zibo 255300, China; 3 College of Fisheries and Life Science, Dalian Ocean University, Dalian 116023, China; 4 Dalian Jinpu New Area Marine Development Affairs Service Center, Dalian 116100, China; 5 State Key Laboratory of North China Crop Improvement and Regulation/Key Laboratory of Hebei Province for Plant Physiology and Molecular Pathology/College of Life Sciences, Hebei Agricultural University, Baoding 071001, China

**Keywords:** PP2C, eelgrass, salt stress, male flower, ABA

## Abstract

Background: Protein Phosphatase 2C (PP2C), a conserved family in plants, plays a crucial role in ABA and MAPK signaling pathways. Its functional diversity provides key mechanisms for plants’ adaptation to environmental changes. However, research on PP2C family members remains significantly underexplored in seagrasses, which are model organisms adapted to complex marine environments. Methods: In this study, we systematically analyzed the PP2C gene family in eelgrass using bioinformatic methods and performed a qPCR experiment to verify the expression of a few members in their response to salt stress. Results: The eelgrass PP2C gene family comprises 52 members, categorized into 13 subfamilies. Most PP2C genes exhibit a differential expression across various organs, with some members showing significant organ specificity. For instance, 12 members are specifically highly expressed in male flowers, suggesting that PP2Cs may function in male flower development. Additionally, four members (*ZosmaPP2C-04*, *ZosmaPP2C-07*, *ZosmaPP2C-15*, and *ZosmaPP2C-18*) in eelgrass are up-regulated under salt stress, with a qPCR confirming their response. The syntenic genes of *ZosmaPP2C-15* and *ZosmaPP2C-18* were identified across multiple species, indicating their evolutionary conservation. Numerous response elements associated with plant hormones and stress were identified within the promoter sequences of eelgrass PP2C genes. Notably, the promoter regions of salt-responsive genes are rich in the ABRE, implying that ABA may participate in regulating the expression of these PP2Cs. Furthermore, the predictive analysis of protein interactions suggests the potential existence of the ABA core signaling module PYL-PP2C-SnRK2 in eelgrass. Conclusions: This study provides a new insight for understanding the biological functions of the PP2C family in eelgrass, which is important for elucidating the mechanisms of its growth, development, and environmental adaptability.

## 1. Introduction

PP2C (Protein Phosphatase Type 2C) is a Mn^2+^- or Mg^2+^-dependent protein serine/threonine phosphatase [1]. In most plants, PP2C represents the largest branch of protein phosphatases (PPs) and plays a crucial role in cell signal transduction by regulating protein activity, interactions, localization, and stability through the removal of phosphate groups from proteins [2].

PP2C has been identified as a core component of the abscisic acid (ABA) signaling pathway in many plant species, acting as a negative regulator. In *Arabidopsis thaliana* (L.) *Heynh*, *ABI1* (*ABA Insensitive 1*) was one of the first PP2Cs identified, and its mutants exhibit an ABA-insensitive phenotype [3]. *ABI2* (*ABA Insensitive 2*) shares functional similarities with *ABI1*, and double mutants of *ABI1* and *ABI2* display an enhanced response to ABA [4]. Additionally, at least five other PP2C members in *A. thaliana*, including *AHG1*, *AHG3*/*PP2CA*, *HAB1*, *HAB2*, and *HAI1*, are involved in the ABA signaling pathway [2]. These PP2Cs belong to the A clade and predominantly regulate downstream events in ABA signaling through interactions with protein kinases SnRK2 [5]. In rice, PP2Cs such as OsPP2C49 [6] and OsPP2C30 [7] modulate ABA responses by interacting with the ABA receptor PYL and protein kinases SnRK2. Similarly, in maize, ZmPP2C-A10 interacts with ZmPYL and ZmSnRK2, participating in ABA signal transduction [8]. Beyond SnRK2, PP2Cs are able to phosphorylate other targets. Research in rice has demonstrated that Ca^2+^/calmodulin-dependent protein kinase (CCaMK), a positive regulator of ABA responses, also interacts with and is inhibited by PP2C [9]. Studies in maize [10,11] and pepper [12] have shown that PP2C can regulate the kinase activity of some members in the MAPK pathway, thereby influencing downstream signal transduction.

Plants are susceptible to environmental factors during their growth and development, and ABA, as a key hormone in response to stress, plays a crucial role in this process. Numerous studies have demonstrated that PP2Cs are involved in the response to abiotic stress through ABA signaling pathways, including cold [13], heat [14], drought [15,16,17], and salinity [18,19,20,21,22,23]. Most of these studies have focused on terrestrial plants but with less attention given to marine plants.

Eelgrass (*Zostera marina*) is a marine angiosperm [24] that can form seagrass beds in both subtidal and intertidal coastal zones. These ecosystems serve as vital habitats, providing food and shelter for a wide range of marine organisms and play a crucial role in maintaining the stability and health of shallow marine environments [25,26]. However, in recent years, seagrass beds have been experiencing a global decline, primarily due to environmental degradation and increasing anthropogenic disturbances [27]. Environmental factors such as fluctuations in temperature and salinity have been shown to negatively affect the survival, morphology, or physiological functions of seagrasses [28,29,30]. Therefore, investigating the molecular mechanisms underlying eelgrass responses to environmental stresses, such as salinity changes, is essential for understanding its adaptive capacity and may provide valuable insights for the conservation and restoration of seagrass ecosystems.

As one of the key gene families involved in salt stress, PP2C proteins have been shown to function as either negative or positive regulatory factors in various species. For example, the *PP2C49* in *A. thaliana* is highly expressed in root vascular tissues and negatively regulates the activity of the ion transporter AtHKT1;1, thereby determining the systemic Na^+^ allocation during salt stress [21]. In rice, *OsPP65* is highly expressed in seedlings and leaves, which negatively regulates osmotic and salt stress tolerances by modulating the ABA and jasmonic acid (JA) signaling pathways, as well as the raffinose family oligosaccharide metabolism pathway [18]. In addition, the protein stability of clade A PP2C family members is regulated by RING-H2 type E3 ligases, which enhance the rice salt tolerance through the degradation of PP2Cs [19]. In contrast the negative roles mentioned above, BpPP2C1 [20] from *Betula platyphylla* and BdPP2CA6 [22] in *Brachypodium distachyon* have been reported to positively regulate plant salt tolerances. *BpPP2C1* overexpressed lines exhibited a significantly enhanced salt tolerance, while *BpPP2C1* knocked-out plants showed an increased sensitivity to salt stress. Moreover, the overexpression of *BdPP2CA6* results in an ABA-hypersensitive phenotype, enhanced stomatal closure, and improved salt tolerance. Our previous transcriptome analysis revealed that several PP2C genes in eelgrass are up-regulated under salt stress [31], indicating their potential involvement in the response to high salinity environments. Thus, this study provides a comprehensive analysis of the PP2C gene family in eelgrass. The salt-responsive expression patterns of several PP2C members were validated through qRT-PCR experiments, and their conservation across species is discussed. Nevertheless, the specific roles of these PP2C members in eelgrass salt tolerance mechanisms require further investigation. Additionally, PP2Cs have been shown to influence plant growth and development, affecting various organs such as flowers [32], leaves [33], fruits [34,35], seeds [36,37,38,39], and cotyledons [40]. Thus, this study explores the differential expression and potential functions of various PP2C members across different organs of eelgrass, laying the groundwork for the further elucidation of the role of PP2C in the developmental regulation of this marine plant.

Notably, our study suggests that the expression of several eelgrass PP2C genes may be regulated by signaling pathways such as ABA, which is consistent with findings in terrestrial plants [2]. However, the underlying molecular mechanisms remain to be confirmed through further experimental validation. In previous studies, the establishment of the core ABA signaling module PYL-PP2C-SnRK2 relied on systematic analyses of genetic transgenic lines, protein–protein interactions, and phosphorylation sites [41]. Characterizing phenotypic changes and transcriptomic responses in transgenic plants helps elucidate the roles of genes in stress adaptation or specific organ development. Since an efficient genetic transformation system for eelgrass has not yet been established, an alternative strategy could involve developing an ectopic expression system, such as introducing eelgrass PP2C genes into model organisms like rice for a functional analysis. These strategies provide valuable references for future investigations into the molecular basis of eelgrass adaptation to marine environments, which will enhance our understanding of stress response mechanisms in seagrasses.

## 2. Materials and Methods

### 2.1. Identification and Characteristics of PP2C Family Members in Eelgrass

The eelgrass genome and gene annotation files were downloaded from the Phytozome V13 (version: *Zostera marina* v3.1; https://phytozome-next.jgi.doe.gov/info/ Zmarina_v3_1, accessed on 29 April 2024). A reference list of 80 *A. thaliana* PP2C genes along with their classification information was obtained from previous research [42]. The protein sequences of *A. thaliana* PP2C were aligned with those of eelgrass using blastp v2.12.0, with an e-value threshold of 1 × 10^−5^. The Hidden Markov Model (HMM) file for the PP2C domain (PF00481) was downloaded from the Pfam database, and hmmscan (HMMER 3.3.2) was employed to search for eelgrass proteins containing this domain. Only those eelgrass genes that showed homology to *A. thaliana* PP2Cs and possessed the PF00481 domain were identified as members of the eelgrass PP2C family.

The gene structures of eelgrass PP2Cs were obtained from genome annotations, with the exons counted. The R package Peptides was utilized to calculate the molecular weight, isoelectric point, and hydrophobicity of the PP2C proteins. Subcellular localization predictions for these proteins were performed using the online tool WoLF PSORT (https://wolfpsort.hgc.jp/, accessed on 18 January 2025).

### 2.2. Phylogenetic and Conserved Motif Analysis

After multiple sequence alignments performed by MAFFT v7.525 using PP2C members from eelgrass and *A. thaliana*, the phylogenetic tree was constructed by FastTree and visualized with the R package ggtree. The conserved motif analysis of eelgrass PP2C was conducted with the MEME v4.11.2 (parameters: -nmotifs 15 -minw 20 -maxw 100) and visualized using the R packages ggplot2.

### 2.3. Chromosomal Localization and Synteny Analysis

The chromosomal location of PP2C members in eelgrass was obtained from gene annotations, and a distribution map was generated using R software v4.3.2. The genome and gene annotation data for *A. thaliana*, rice, and maize were downloaded from Ensembl Plant. Homologous genes within eelgrass (parameters: -evalue 1 × 10^−10^ -qcov_hsp_perc 50, identity >= 30) or between eelgrass and *A. thaliana*, rice, maize, and three other seagrasses *Cymodocea nodosa*, *Posidonia oceanica*, and *Thalassia testudinum* (parameters: -evalue 1 × 10^−5^ -max_target_seqs 5) were identified by blastp and then used for synteny analysis by MCScanX.

### 2.4. Expression Analysis of PP2C Members

The expression profile data of eelgrass PP2Cs from leaf, root, seed, female flower, and male flower were obtained from our published studies [31]. Genes that were positively correlated with these PP2Cs at transcriptional level were collected (Pearson coefficient ≥ 0.9). The read counts for all genes in the samples were calculated using featureCounts and we performed differential expression analysis between organs with DESeq2 (FDR ≤ 0.05 and fold change ≥ 2), which identified a set of PP2C members with organ-specific high expression. GO enrichment analysis was conducted with the R package ClusterProfiler on a gene set associated with high expression of PP2Cs in male flowers and leaves. In addition, we visualized the expression levels of four PP2C members’ responsive to salt stress which were identified from published studies.

### 2.5. qRT-PCR Experiment

The SteadyPure Plant RNA Extraction Kit (Accurate Biotechnology, Changsha, Hunan, China) was utilized for mRNA extraction. The Evo M-MLV Reverse Transcription Kit (Accurate Biotechnology, Changsha, Hunan, China) was utilized for reverse transcription of RNA. Quantitative real-time PCR (qRT-PCR) experiments were carried out using 7500 Real-Time PCR equipment (ABI, Foster city, CA, USA), with the following thermal cycling conditions: 95 °C for 30 s, followed by 40 cycles at 95 °C for 5 s and 60 °C for 30 s. In total, the expressions of four PP2Cs in response to salt stress were verified. The primers for these genes are listed in Table S1. A ubiquitin gene served as the reference gene for normalization, and relative expression changes were calculated using the 2^−ΔΔCt^ method. The statistical analysis was performed using the function t.test in R software v4.3.2.

### 2.6. Cis-Acting Regulatory Elements Analysis

The 1000 bp upstream sequences of the PP2C genes from eelgrass were obtained based on genome annotations and used for identifying cis-acting regulatory elements by the online tool PlantCARE. The predicted cis-acting regulatory elements were then summarized and visualized using the R packages ggplot2 and ComplexHeatmap.

### 2.7. Prediction of PYL-PP2C-SnRK2 Module in Eelgrass

The list of PYL and SnRK2 family genes in *A. thaliana* was obtained from the Tair database (https://www.arabidopsis.org/, accessed on 3 February 2025). The protein sequences of *A. thaliana* PYL and SnRK2 were compared with the protein sequences of eelgrass using blastp (e-value ≤ 1 × 10^−5^; identity ≥ 40). HMM files for the key domains Polyketide_cyc2 (PF10604) of PYL and Pkinase (PF00069) of SnRK2 were downloaded from the Pfam database and used in hmmscan to search for eelgrass proteins containing these domains. Only eelgrass genes that were homologous to *A. thaliana* genes and possessed the key protein domains were identified as candidate family members. The eelgrass SnRK2 family members were further refined by constructing a phylogenetic tree. Ultimately, five PYL and four SnRK2 members were identified in eelgrass. Potential interactions between eelgrass PYL, PP2C, and SnRK2 were predicted using the STRING database (version 12.0), with *A. thaliana*, rice, and maize proteins as organism models (the active interaction sources were set to "Experiments", which show only experimentally determined interactions). The protein interaction network was visualized using Cytoscape software v3.10.2.

## 3. Results

### 3.1. Genome-Wide Identification of Eelgrass PP2Cs

**Figure 1:**
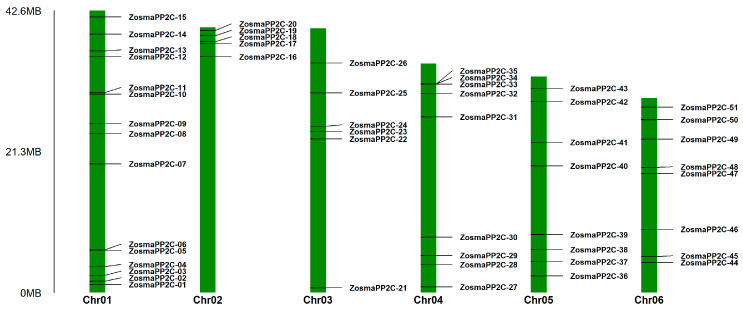
Chromosomal localization of eelgrass PP2Cs.

A total of 52 PP2C family members were identified in the eelgrass genome and were sequentially named according to their chromosomal locations as *ZosmaPP2C-01* to *ZosmaPP2C-52* (Table 1; Figure 1). The number of amino acid residues in the proteins encoded by these genes varies significantly, with ZosmaPP2C-38 having the fewest (71 aa) and ZosmaPP2C-39 having the most (1076 aa). The molecular weights of these proteins range from 7.83 to 121.35 kDa, and their theoretical isoelectric points (pI) range from 4.41 to 9.26. Most eelgrass PP2Cs are acidic proteins, with 36 members (69%) having a pI less than seven, while the others are basic proteins. Additionally, the majority of these proteins are hydrophilic, with only ZosmaPP2C-03 and ZosmaPP2C-38 being hydrophobic. The prediction results of the subcellular localization indicate that eelgrass PP2C proteins are mainly localized in the chloroplast (36.5%), cytoplasm (26.9%), and nucleus (25%).

### 3.2. Phylogenetic Analysis and Classification of Eelgrass PP2Cs

**Figure 2:**
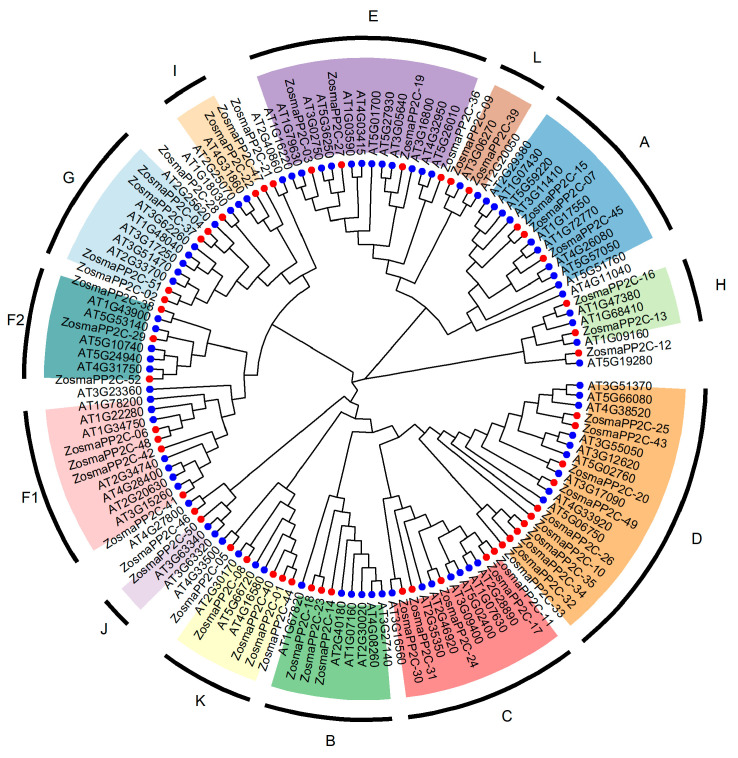
Phylogenetic tree of PP2Cs in eelgrass and *A. thaliana*. The colors represent different groups of PP2Cs. Blue circles represent *A. thaliana* genes, and red circles represent eelgrass genes.

A phylogenetic tree was constructed using PP2C protein sequences from eelgrass and *A. thaliana*. Based on previous classifications of *A. thaliana* PP2C, 47 eelgrass PP2Cs were grouped into 13 subfamilies (Figure 2). Subfamily D contained the most eelgrass PP2Cs, with a total of 10 members, while subfamily J had only 1 member. Additionally, five members (ZosmaPP2C-05, ZosmaPP2C-12, ZosmaPP2C-21, ZosmaPP2C-28, and ZosmaPP2C-46) were not classified into any subfamily, as they are homologous to unclassified *A. thaliana* PP2Cs. However, the phylogenetic tree indicates that they are similar to members of subfamilies K, H, I, G, and J, respectively.

### 3.3. The Gene Structure of Eelgrass PP2Cs

The eelgrass PP2C family members exhibit a range of exon numbers from 1 to 24 (Table 1), with only four members lacking introns. A total of 46 PP2C members have 10 or fewer exons, accounting for 88.5%. The most common among them are those with four exons, totaling 19 members. Except for *ZosmaPP2C-12*, *ZosmaPP2C-33* and *ZosmaPP2C-50*, most members have gene lengths under 10 kb. *ZosmaPP2C-50* has 20 exons and a gene length of 73 kb, while *ZosmaPP2C-33*, with the highest number of exons, has a gene length of nearly 17 kb.

### 3.4. Protein Domain and Conserved Motifs of Eelgrass PP2Cs

**Figure 3:**
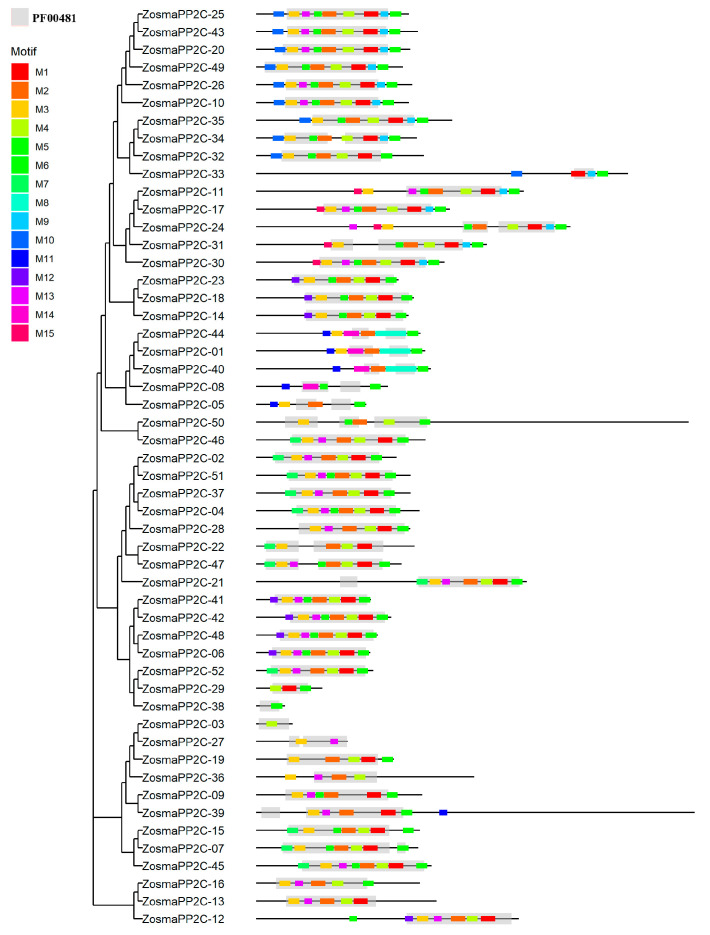
Conserved protein motifs of eelgrass PP2Cs.

All PP2C proteins in eelgrass contain the conserved PF00481 domain. In order to further explore the structural characteristics of these proteins, a conserved motif analysis was performed on their sequences. (Figure 3). The results revealed that most PP2C proteins (48 in total) contain more than three conserved motifs. Among the fifteen identified motifs, seven motifs (Motif1-6 and Motif13) are present in more than half of the PP2C members. The distribution of conserved motifs is similar within many subfamilies. For instance, subfamily A contains motifs M1–M7, subfamily B contains motifs M1–M6 and motif M12, most of subfamily C includes motifs M1–M6, M9, M13, and M15, and most of subfamily D comprises motifs M1–M6, M9, and M10. Furthermore, subfamily F1 includes motifs M1-M6, M12, and M13, while subfamily G features motifs M1–M5, M7, and M13. Notably, some motifs are exclusive to specific subfamilies, such as motifs M8 and M14 in subfamily K and motif M15 in subfamily C. These differences in the conserved motif distribution may indicate functional distinctions among the subfamilies.

### 3.5. Chromosomal Localization and Synteny Analysis of Eelgrass PP2Cs

**Figure 4:**
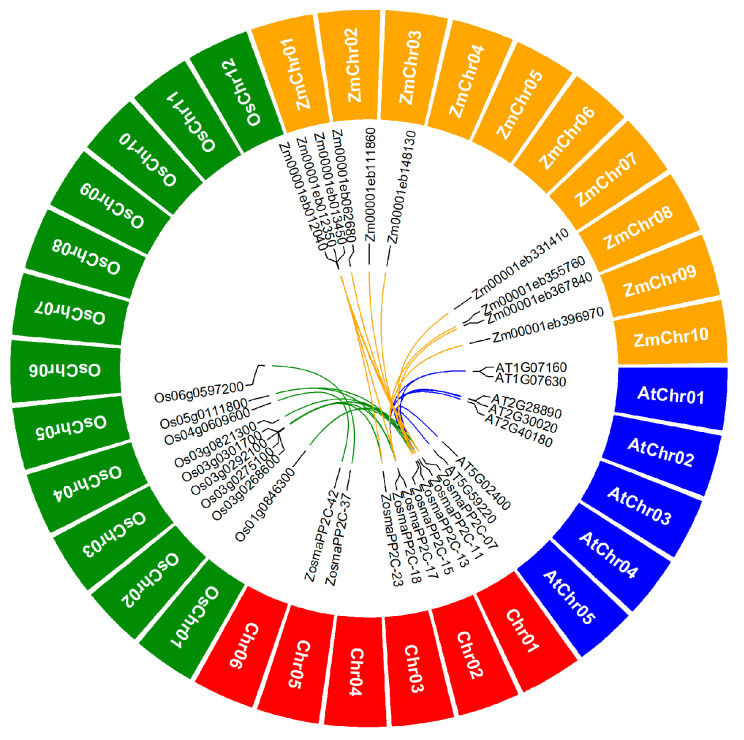
Synteny analysis of eelgrass PP2Cs.

The genomic location of eelgrass PP2Cs reveals that, with the exception of *ZosmaPP2C-52* which is located on a scaffold (Table 1), the remaining members of the PP2Cs are unevenly distributed across six chromosomes (Figure 1). Chromosome 1 hosts the highest number of PP2C members, totaling 15, while Chromosome 2 contains the fewest, with only 5 members. The synteny analysis indicates the presence of seven pairs of collinear PP2C members within the eelgrass (Table S2). Notably, five of these collinear pairs originate from the same subfamily, including subfamily B (*ZosmaPP2C-14*, *ZosmaPP2C-18* and *ZosmaPP2C-23*), subfamily C (*ZosmaPP2C-11* and *ZosmaPP2C-17*), subfamily F1 (*ZosmaPP2C-06* and *ZosmaPP2C-48*), and subfamily G (*ZosmaPP2C-04* and *ZosmaPP2C-37*). Furthermore, the synteny analysis between the eelgrass and the other three species *A. thaliana*, rice, and maize identifies a total of 31 collinear gene pairs which include nine eelgrass PP2Cs (Figure 4; Table S2). Among these, *ZosmaPP2C-11*, *ZosmaPP2C-18*, and *ZosmaPP2C-15* show collinear gene pairs across all three species, indicating the higher conservation of these members. Additionally, we observed more collinear gene pairs between the eelgrass and other seagrass species. Specifically, 50, 39 and 36 collinear PP2C gene pairs were identified when comparing eelgrass with *Cymodocea nodosa*, *Posidonia oceanica* and *Thalassia testudinum*, respectively, which cover 42 PP2C members from the eelgrass in total. This finding suggests a closer phylogenetic relationship among these seagrass species (Table S2).

### 3.6. The Expression of Eelgrass PP2Cs in Different Tissues and in Response to Salt Stress

**Figure 5:**
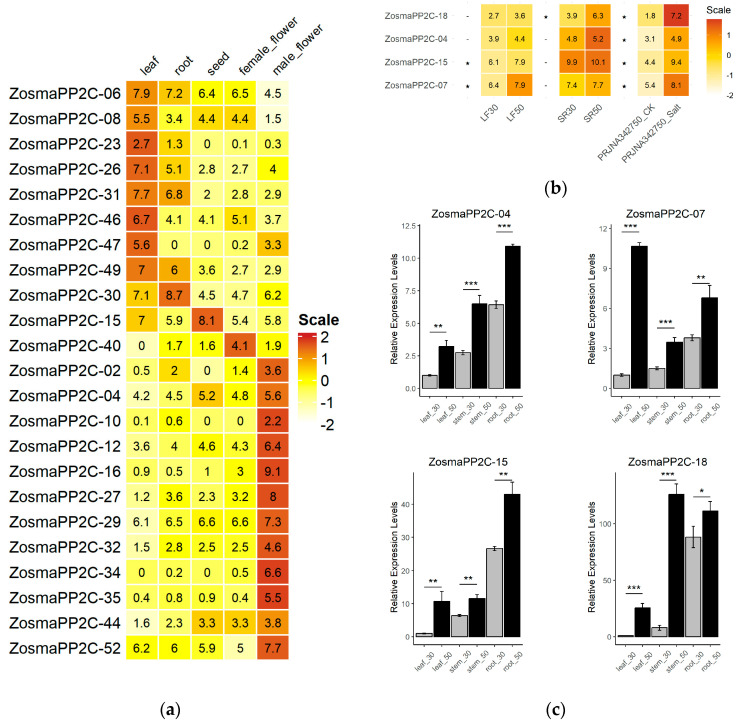
The expression analysis of eelgrass PP2Cs. (**a**) Expression profiles of organ-specific PP2Cs in eelgrass; (**b**) expression profiles of salt-responsive PP2Cs in eelgrass; and (**c**) the validation of 4 eelgrass PP2Cs in response to the salt stress by the qPCR. Three tissues (leaf, stem, root) were analyzed under two salinity conditions: 30 (control) and 50 (high salinity). The star marks on statistical charts represent the statistical significance: “*” stands for *p*-value < 0.05, “**” stands for *p*-value < 0.01, “***” stands for *p*-value < 0.001.

The PP2C genes in eelgrass exhibit varying levels of expression across different organs. Some members, such as *ZosmaPP2C-22* and *ZosmaPP2C-45*, display a constitutive expression pattern. The abundance of these two genes does not show significant differences among organs, and their expression levels (FPKM) are greater than 10 across all samples, with a coefficient of variation (CV) < 0.5. By contrast, many other members in the eelgrass exhibit distinct organ-specific expression patterns (Figure 5a). For instance, 12 genes, including *ZosmaPP2C-02*, *ZosmaPP2C-04*, *ZosmaPP2C-10*, *ZosmaPP2C-12*, *ZosmaPP2C-16*, *ZosmaPP2C-27*, *ZosmaPP2C-29*, *ZosmaPP2C-32*, *ZosmaPP2C-34*, *ZosmaPP2C-35*, *ZosmaPP2C-44*, and *ZosmaPP2C-52*, are highly expressed in male flowers. Eight genes, such as *ZosmaPP2C-06*, *ZosmaPP2C-08*, *ZosmaPP2C-23*, *ZosmaPP2C-26*, *ZosmaPP2C-31*, *ZosmaPP2C-46*, *ZosmaPP2C-47* and *ZosmaPP2C-49*, show high expression levels in leaves. Additionally, a specific high expression is observed for *ZosmaPP2C-40* in female flowers, *ZosmaPP2C-30* in roots, and *ZosmaPP2C-15* in seeds. Moreover, genes associated with organ-specific PP2C expression are enriched in particular biological processes, such as pollination in male flowers (Figure S1) and photosynthesis in the leaf (Figure S2). These findings suggest that different PP2C members may play unique roles in specific organs of eelgrass.

Furthermore, transcriptome comparisons revealed that four PP2C members, including *ZosmaPP2C-07* and *ZosmaPP2C-15* from the A subfamily, *ZosmaPP2C-18* from the B subfamily, and *ZosmaPP2C-04* from the G subfamily, exhibit an up-regulated expression under salt stress (Figure 5b). The transcriptional response of these genes has been confirmed by the qPCR analysis (Figure 5c).

### 3.7. Cis-Acting Regulatory Element of Eelgrass PP2Cs on Promoter Region

**Figure 6:**
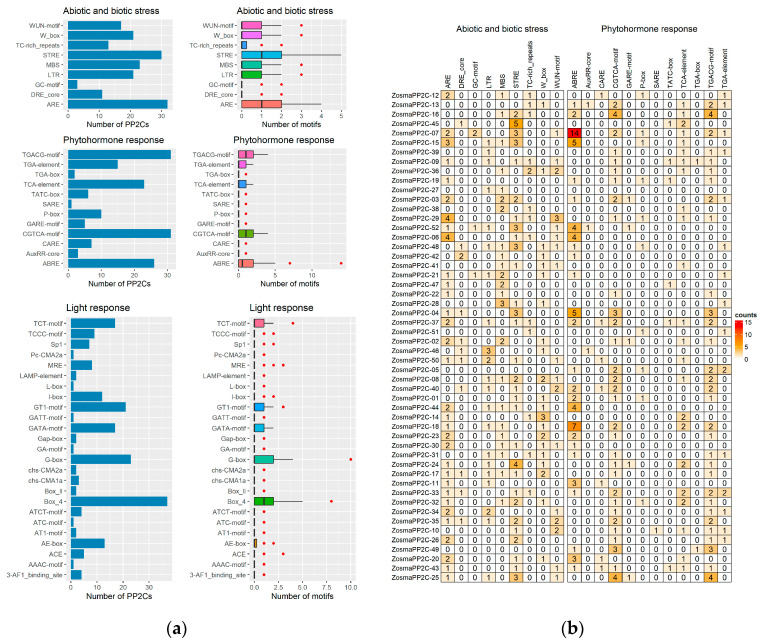
The cis-acting regulatory element of eelgrass PP2Cs on the promoter region. (**a**) Bar plots showing the number of PP2Cs that contain a specific motif and box plots showing the number of specific motifs on the promoter of PP2C genes. (**b**) The count of specific motifs on the promoter region of each PP2C gene.

The promoter regions of most eelgrass PP2C genes contain cis-acting regulatory elements associated with stress responses or hormone signaling (Figure 6a). Specifically, more than half of these promoters include stress-responsive elements, such as ARE (61.5%) and STRE (57.7%), as well as ABA-responsive elements, ABRE (50%), and JA-responsive elements, the CGTCA-motif and TGACG-motif (59.6%). These findings suggest that eelgrass PP2C genes may be induced by stress conditions and the hormones ABA and JA. Notably, the promoters of genes *ZosmaPP2C-07*, *ZosmaPP2C-15*, *ZosmaPP2C-18*, and *ZosmaPP2C-04*, which are significantly up-regulated under salt stress, contain a higher number of ABRE elements, with 14, 5, 7 and 5 elements, respectively (Figure 6b). This indicates that ABA signaling may mediate the response of eelgrass to high salinity through these genes. Additionally, the promoters of most eelgrass PP2C genes are rich in various light-responsive elements, such as Box_4, G-box, and GT1-motif, suggesting a potential regulation by light (Figure 6a).

### 3.8. Prediction of PP2C-Interacting Proteins in Eelgrass

**Figure 7:**
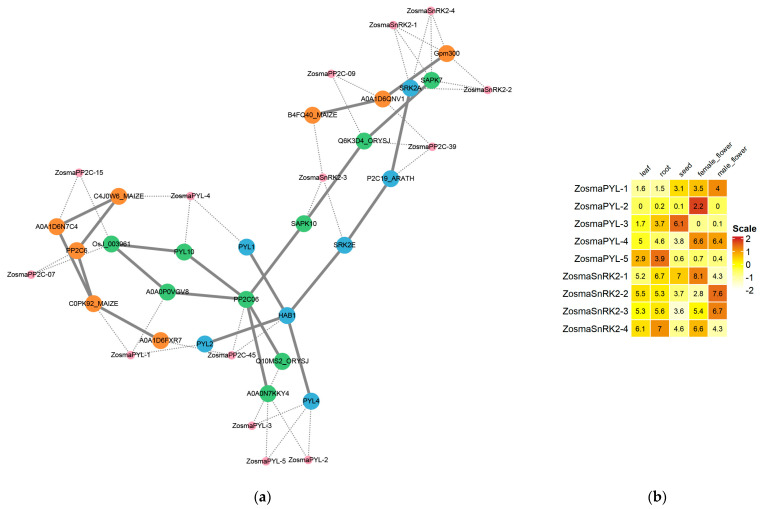
Potential PYL-PP2C-SnRK2 modules in eelgrass. (**a**) The prediction of PP2C-interacting proteins in eelgrass. The blue dots represent genes of *A. thaliana*, the green dots represent genes of rice, the orange dots represent genes of maize, and the pink represent genes of eelgrass. Solid lines represent the interactions documented in the STRING database, whereas dashed lines illustrate the mapping relationships between the eelgrass genes and those recorded in the database. (**b**) Organ-specific expression patterns of PYL and SnRK2 family members in eelgrass.

In terrestrial plants, it is known that PYL-PP2C-SnRK2 constitutes a crucial regulatory module in the ABA signaling pathway, and there is physical interactions among these components. Thus, we identified the SnRK2 and PYL in eelgrass and predicted potential interactions between members of these two families and the PP2C (Figure 7a). The results indicate that ZosmaPP2C-45 likely interacts with ZosmaSnRK2-3 and multiple PYL proteins; ZosmaPP2C-15 and ZosmaPP2C-07 may interact with ZosmaPYL1/4; and ZosmaPP2C-09 and ZosmaPP2C-39 potentially interact with several ZosmaSnRK proteins.

## 4. Discussion

In this study, we conducted a systematic bioinformatics analysis of the PP2C gene family within the eelgrass genome. The findings indicate that there are 52 PP2C genes in eelgrass, which is fewer compared to the numbers found in *A. thaliana* and rice. A further examination revealed that these PP2C genes can be categorized into 13 subfamilies (labeled A to L, with F divided into F1 and F2), which aligns with the classification observed in *A. thaliana*. Additionally, members of the same PP2C subfamily display similar conserved motifs in protein sequences, indicating their close evolutionary relationships and providing more evidence for the constructed phylogenetic tree.

The expression analysis results show that PP2C genes are widely expressed in various organs of eelgrass. Some genes exhibit a constitutive expression pattern, while others show organ specificity. Research in rice [32] indicates that the PP2C family member DCW11 mediates mitochondrial signaling transduction during pollen germination. This gene is highly expressed in anthers and down-regulated in the anthers of CW-type male sterile lines. Knocking down *DCW11* in normal plants can lead to a loss of seed set fertility. We found that several PP2C genes in eelgrass exhibit high expressions, specifically in male flowers, indicating their potential roles in male flower development and suggesting the possibility of functional redundancy among these genes.

The promoter analysis indicates that the upstream sequences of most eelgrass PP2C members contain ABA and JA response elements, suggesting that the eelgrass PP2C expression may be induced by ABA and JA signals. In addition, the promoter sequences of the eelgrass PP2C include various stress-responsive elements associated with anaerobic conditions, low temperatures, drought, and wounding, implying that multiple stress signals can regulate the transcription of PP2Cs in eelgrass. Previous studies have demonstrated that the expression of some PP2C genes is up-regulated under salt stress in terrestrial plants such as *A. thaliana* [43], rice [44], wheat [45], and cotton [46]. In eelgrass, a few PP2Cs also exhibit a similar response pattern under salt stress, suggesting that the regulatory mechanisms of some PP2Cs at the transcriptional level might be conserved across different species. This speculation is further supported by the syntenic analysis: *ZosmaPP2C-15* and *ZosmaPP2C-18*, which are significantly up-regulated under salt stress, both have collinear gene pairs in *A. thaliana*, rice, and maize. Previous studies have shown that *HAI1* (*AT5G59220*), a homolog of *ZosmaPP2C-15*, functions as a negative regulator of osmotic adjustment [47] and participates in the ABA signal transduction [48]. Similarly, *PP2C5* (*AT2G40180*), a homolog of *ZosmaPP2C-18*, regulates seed germination, stomatal closure, and ABA-induced gene expression [49]. Further research is required to elucidate the regulatory factors mediating this response in eelgrass.

The expression of some PP2Cs in terrestrial plants could be influenced by ABA [2]. Our study indicates that ABA may play a role in the response in eelgrass. Because the promoter regions of four PP2C members whose expression is induced under salt stress are enriched with more ABRE elements compared to other PP2C genes. ABRE (ABA-responsive element) is a key cis-acting regulatory element that mediates ABA-responsive gene expression [50] and is critical for plant adaptations to abiotic stresses, including salinity and drought [51]. Some ABA-inducible genes harbor clustered ABREs that function cooperatively [52,53]. Members of the bZIP transcription factor family act as ABA-responsive element-binding factors (ABFs) that specifically recognize ABREs [50]. The molecular mechanism by which ABA regulates ABFs has been well characterized in terrestrial plants [50]: ABA inhibits the phosphatase activity of PP2Cs through its receptor PYL, thereby releasing the inhibition of SnRK2 kinases by PP2Cs. Activated SnRK2 then phosphorylates ABFs, leading to the up-regulation of downstream stress-responsive genes. Moreover, ABFs have been shown to bind to the promoters of multiple PP2C genes, inducing their rapid expression under abiotic stress and forming a negative feedback loop that helps maintain the dynamic balance in the ABA signaling pathway [54]. Based on these findings, we propose that the observed up-regulation of PP2C genes in eelgrass under salt stress may involve a similar regulatory mechanism.

Our study suggests that the key regulatory module of the ABA signaling pathway, PYL-PP2C-SnRK2, is likely present in eelgrass. We observed distinct organ-specific expression patterns of PYL and SnRK2 family members in eelgrass (Figure 7b). For instance, *ZosmaPYL-3* is highly expressed in seeds, whereas *ZosmaPYL-2* and *ZosmaSnRK2-1* are predominantly expressed in female flowers. In male flowers, *ZosmaPYL-1*, *ZosmaSnRK2-2*, and *ZosmaSnRK2-3* show high expression levels. Based on expression similarities and potential protein interactions, we hypothesize that ZosmaPYL-1, ZosmaSnRK2-3, and the constitutively expressed ZosmaPP2C-45 may function as an ABA signaling module during male flower development. Furthermore, although the expression of eelgrass PYL and SnRK2 family members is not affected by salt stress, ZosmaPP2C-15 and ZosmaPP2C-07, which are up-regulated at a high salinity, potentially interact with two PYLs (ZosmaPYL1 and ZosmaPYL4). This implies that ABA signaling may also regulate the function of PP2C at the protein level under salt stress.

In terrestrial plants, ABA rapidly accumulates under salt stress conditions [55]. However, there is currently no experimental evidence to confirm whether a similar ABA accumulation and response mechanism exists in marine plants. To address this question, we plan to quantify ABA levels in various tissues of eelgrass under both high-salinity and control conditions in future studies. This will help elucidate the dynamic changes in ABA during salt stress responses in eelgrass and its potential regulatory functions. This research will contribute to a deeper understanding of hormonal regulatory mechanisms in marine plants under abiotic stress.

## 5. Conclusions

This study provides a comprehensive analysis of the PP2C gene family in eelgrass and explores the potential roles of these genes in its plant development and stress response. As shown in the results, many PP2C members are conserved across species, and the PP2C-mediated ABA signal transduction is likely involved in eelgrass’s response to salt stress. These findings lay a foundation for understanding the biological functions of PP2C genes and provide new insights into the mechanisms that enable eelgrass to adapt to environmental changes. In the future, we plan to further investigate the roles of PP2C-related core ABA modules in eelgrass during stress responses and organ development. For example, yeast two-hybrid assays can be employed to identify interacting partners of PP2Cs, such as PYL and SnRK2. Additionally, by constructing ectopic transgenic plants, the functions of specific PP2C genes will be determined under stress conditions or in the development of particular organs. These studies will enhance our understanding of eelgrass’s adaptation to the marine environment.

## Figures and Tables

**Table 1:** Genome-wide identification of eelgrass PP2Cs.

Gene_ID	Gene_Name	Genomic Position	Exon Number	Amino Acid	Mw	pI	Hydropathicity	Location
Zosma02g21130	ZosmaPP2C-17	Chr02:37645131-37646558(+)	1	476	52,968.61	5.22	−0.345	nucl
Zosma02g23620	ZosmaPP2C-19	Chr02:38842882-38844276(+)	4	338	37,679.66	6.21	−0.367	nucl
Zosma02g18050	ZosmaPP2C-16	Chr02:35640155-35642265(+)	9	402	43,424.27	4.74	−0.211	cyto
Zosma02g25420	ZosmaPP2C-20	Chr02:39635934-39637992(+)	4	378	41,661.17	6.25	−0.273	cyto
Zosma02g21670	ZosmaPP2C-18	Chr02:37935610-37936910(+)	2	388	42,198.18	5.63	−0.339	chlo
Zosma01g02300	ZosmaPP2C-02	Chr01:1723172-1724206(+)	1	345	38,260.02	5.07	−0.365	nucl
Zosma01g03800	ZosmaPP2C-03	Chr01:2596913-2597182(−)	1	90	9874.10	4.56	0.046	cyto
Zosma01g18970	ZosmaPP2C-08	Chr01:24010873-24012921(−)	10	323	34,955.8	5.65	−0.116	chlo
Zosma01g36660	ZosmaPP2C-14	Chr01:39054616-39056106(−)	3	374	40,638.99	5.98	−0.317	chlo
Zosma01g25210	ZosmaPP2C-11	Chr01:30232032-30234173(−)	3	657	72,553.42	5.6	−0.432	nucl
Zosma01g33240	ZosmaPP2C-13	Chr01:36512517-36515120(+)	7	443	47,269.78	5.86	−0.185	nucl
Zosma01g06030	ZosmaPP2C-04	Chr01:3891751-3894444(−)	4	401	43,330.71	4.72	−0.154	chlo
Zosma01g16060	ZosmaPP2C-07	Chr01:19447211-19448485(−)	2	398	43,151.96	5.6	−0.27	nucl
Zosma01g41250	ZosmaPP2C-15	Chr01:41671572-41673020(+)	4	402	43,831.35	7.2	−0.414	cyto
Zosma01g09130	ZosmaPP2C-05	Chr01:6297640-6298838(−)	6	271	29,309.02	5.04	−0.167	cyto
Zosma01g24910	ZosmaPP2C-10	Chr01:30001865-30003225(+)	4	375	42,879.38	8.61	−0.281	nucl
Zosma01g20530	ZosmaPP2C-09	Chr01:25525319-25526706(−)	3	408	44,707.5	4.47	−0.335	cyto
Zosma01g01360	ZosmaPP2C-01	Chr01:1226328-1228062(+)	4	415	44,827.16	7.19	−0.259	chlo
Zosma01g32210	ZosmaPP2C-12	Chr01:35669026-35687172(−)	14	645	71,138.92	5.88	−0.214	chlo
Zosma01g09260	ZosmaPP2C-06	Chr01:6435541-6436897(−)	5	280	30,367.56	9.23	−0.431	cyto
Zosma04g06830	ZosmaPP2C-29	Chr04:5613604-5614375(−)	4	162	17,801.05	4.61	−0.254	cyto
Zosma04g16720	ZosmaPP2C-31	Chr04:26553016-26554963(−)	4	567	62,401.61	6.68	−0.493	cyto
Zosma04g22800	ZosmaPP2C-34	Chr04:31522865-31524049(+)	1	395	45,150.45	6.31	−0.536	nucl
Zosma04g22790	ZosmaPP2C-33	Chr04:31505790-31522743(+)	24	913	103,337.17	6.99	−0.146	chlo
Zosma04g21550	ZosmaPP2C-32	Chr04:30094279-30095776(−)	4	412	46,772.25	6.26	−0.276	chlo
Zosma04g09260	ZosmaPP2C-30	Chr04:8351211-8352819(−)	3	462	50,534.86	4.67	-0.214	nucl
Zosma04g05430	ZosmaPP2C-28	Chr04:4230755-4234336(+)	10	378	41,317.95	8.04	−0.365	cyto
Zosma04g00980	ZosmaPP2C-27	Chr04:863428-864629(−)	3	225	25,499.52	6.25	−0.622	chlo
Zosma04g22810	ZosmaPP2C-35	Chr04:31524979-31526648(−)	4	481	54,856.74	9.2	−0.236	cysk
Zosma06g09020	ZosmaPP2C-45	Chr06:5412181-5413730(−)	4	431	47,071.05	5.03	−0.206	chlo
Zosma06g12670	ZosmaPP2C-46	Chr06:9544529-9548632(−)	11	416	45,739.09	7.73	−0.11	chlo
Zosma06g07830	ZosmaPP2C-44	Chr06:4547808-4551544(+)	4	404	43,622.19	8.05	−0.141	chlo
Zosma06g28610	ZosmaPP2C-51	Chr06:28014762-28016144(+)	4	379	41,245.42	5.97	−0.351	chlo
Zosma06g22190	ZosmaPP2C-49	Chr06:23169549-23170715(+)	2	360	40,348.93	7.77	−0.358	chlo
Zosma06g16930	ZosmaPP2C-48	Chr06:18926155-18927551(+)	5	299	32,721.32	7.85	−0.325	cyto
Zosma06g26550	ZosmaPP2C-50	Chr06:26097002-26170395(+)	20	1062	121,353.82	6.98	−0.373	chlo
Zosma06g15960	ZosmaPP2C-47	Chr06:18007315-18009251(+)	10	357	39,322.92	8.01	−0.322	cysk
Zosma154g00070	ZosmaPP2C-52	scaffold_154:65907-68395(−)	8	288	30,882.72	5.36	−0.189	cyto
Zosma05g21020	ZosmaPP2C-41	Chr05:22707710-22709375(−)	5	282	30,696.04	7.84	−0.275	cyto
Zosma05g31280	ZosmaPP2C-43	Chr05:30867806-30869625(+)	4	397	43,802.91	8.94	−0.291	chlo
Zosma05g04480	ZosmaPP2C-36	Chr05:2491529-2498534(+)	10	535	58,109.03	4.58	−0.25	cyto
Zosma05g18030	ZosmaPP2C-40	Chr05:19136954-19138522(−)	4	429	46,362.18	6.59	−0.176	chlo
Zosma05g07410	ZosmaPP2C-37	Chr05:4635552-4637065(−)	4	379	41,150.38	5.09	−0.192	cysk
Zosma05g28040	ZosmaPP2C-42	Chr05:28833135-28834253(+)	3	332	36,036.37	9.26	−0.489	nucl
Zosma05g10930	ZosmaPP2C-38	Chr05:6485671-6486054(+)	3	71	7833.00	4.41	0.176	extr
Zosma05g13440	ZosmaPP2C-39	Chr05:8751625-8758541(−)	15	1076	120,748.65	5.07	−0.363	nucl
Zosma03g12800	ZosmaPP2C-22	Chr03:23217337-23220806(+)	10	389	42,278.27	5.03	−0.406	nucl
Zosma03g27860	ZosmaPP2C-26	Chr03:34707412-34709175(−)	4	383	42,883.17	8.13	−0.241	chlo
Zosma03g14770	ZosmaPP2C-24	Chr03:25132638-25135189(−)	4	772	85,688.82	5.7	−0.504	nucl
Zosma03g00750	ZosmaPP2C-21	Chr03:720520-724643(+)	11	665	74,159.99	6.03	−0.23	mito
Zosma03g13840	ZosmaPP2C-23	Chr03:24352649-24353861(−)	3	350	37,628.48	5.33	−0.181	extr
Zosma03g21390	ZosmaPP2C-25	Chr03:30186101-30189998(+)	4	375	42,268.25	9.11	−0.328	chlo

## Data Availability

The original contributions presented in the study are included in the article/Supplementary Materials. Further inquiries can be directed to the corresponding author.

## Supplementary Materials

The following supporting information can be downloaded at https://www.mdpi.com/article/10.3390/genes16060657/s1, Table S1: Primers used in qPCR experiment; Table S2: Result of synteny analysis; Figure S1: GO terms enriched for genes associated with the PP2Cs that show specific high expression in male flower; Figure S2: GO terms enriched for genes associated with the PP2Cs that show specific high expression in leaves.

## Abbreviations

The following abbreviations are used in this manuscript:

PP2C	Protein Phosphatase 2C
ABA	abscisic acid
ABRE	Abscisic Acid Response Element
PYL	Pyrabactin resistance 1-like
SnRK2	SNF1-related protein kinase 2
nucl	nucleus
cyto	cytoplasm
chlo	chloroplast
mito	mitochondria
cysk	cytoskeleton
extr	extracellular
