# Supplementary material for: Genome-Wide Identification and Expression Analysis of PP2C Gene Family in Eelgrass
Source: Genes (Basel). 2025 May 29;16(6):657. doi: 10.3390/genes16060657 (PMC12193245; doi:10.3390/genes16060657)

Supplementary Materials

Table S1. Primers used in qPCR experiment.

**Primer_name**	**Primer_Sequence**
PP2C-04-F	GTCCCAAGGTTCCGTGTCAATAG
PP2C-04-R	TCATCTCCTCCTCCAAAGCCATC
PP2C-07-F	GGAGAAGGAAGGAGATGGAGGATG
PP2C-07-R	CAAACACGGCGAAGAAATGGTAAC
PP2C-15-F	TCGGTTCAACAGCGGTGGTAG
PP2C-15-R	GAACGGCGACTCCTCCTCTG
PP2C-18-F	GCCAGCACCACCAATTCTACC
PP2C-18-R	ACCTCTTTCCCTCATCCACATCC
Ubiquitin-F	TTCGCGTGAAGTGCAATGGAGAC
Ubiquitin-R	CTCCGCTCTGGTCCCAGTCTG

Table S2. Result of synteny analysis.

Synteny Analysis	Collinear PP2C member 1	Collinear PP2C member 2
within eelgrass	ZosmaPP2C-09	ZosmaPP2C-18
within eelgrass	ZosmaPP2C-11	ZosmaPP2C-17
within eelgrass	ZosmaPP2C-14	ZosmaPP2C-23
within eelgrass	ZosmaPP2C-04	ZosmaPP2C-37
within eelgrass	ZosmaPP2C-06	ZosmaPP2C-48
within eelgrass	ZosmaPP2C-18	ZosmaPP2C-23
within eelgrass	ZosmaPP2C-21	ZosmaPP2C-45
between eelgrass and *A. thaliana*	AT1G07630	ZosmaPP2C-11
between eelgrass and *A. thaliana*	AT1G07160	ZosmaPP2C-18
between eelgrass and *A. thaliana*	AT2G28890	ZosmaPP2C-11
between eelgrass and *A. thaliana*	AT2G40180	ZosmaPP2C-18
between eelgrass and *A. thaliana*	AT2G30020	ZosmaPP2C-18
between eelgrass and *A. thaliana*	AT5G59220	ZosmaPP2C-15
between eelgrass and *A. thaliana*	AT5G02400	ZosmaPP2C-11
between eelgrass and rice	Os01g0846300	ZosmaPP2C-15
between eelgrass and rice	Os01g0846300	ZosmaPP2C-07
between eelgrass and rice	Os03g0268600	ZosmaPP2C-07
between eelgrass and rice	Os03g0821300	ZosmaPP2C-11
between eelgrass and rice	Os03g0275100	ZosmaPP2C-11
between eelgrass and rice	Os03g0301700	ZosmaPP2C-13
between eelgrass and rice	Os03g0292100	ZosmaPP2C-18
between eelgrass and rice	Os03g0292100	ZosmaPP2C-23
between eelgrass and rice	Os04g0609600	ZosmaPP2C-42
between eelgrass and rice	Os05g0111800	ZosmaPP2C-11
between eelgrass and rice	Os05g0111800	ZosmaPP2C-17
between eelgrass and rice	Os06g0597200	ZosmaPP2C-37
between eelgrass and maize	Zm00001eb012040	ZosmaPP2C-07
between eelgrass and maize	Zm00001eb062680	ZosmaPP2C-11
between eelgrass and maize	Zm00001eb012350	ZosmaPP2C-11
between eelgrass and maize	Zm00001eb012350	ZosmaPP2C-17
between eelgrass and maize	Zm00001eb013450	ZosmaPP2C-18
between eelgrass and maize	Zm00001eb013450	ZosmaPP2C-23
between eelgrass and maize	Zm00001eb111860	ZosmaPP2C-13
between eelgrass and maize	Zm00001eb148130	ZosmaPP2C-15
between eelgrass and maize	Zm00001eb331410	ZosmaPP2C-13
between eelgrass and maize	Zm00001eb367840	ZosmaPP2C-15
between eelgrass and maize	Zm00001eb355760	ZosmaPP2C-17
between eelgrass and maize	Zm00001eb396970	ZosmaPP2C-18

Between eelgrass and *Cymodocea nodosa*

Collinear PP2C member 1	Collinear PP2C member 2
Cymno02g02660	ZosmaPP2C-06
Cymno02g19010	ZosmaPP2C-07
Cymno02g17920	ZosmaPP2C-13
Cymno03g09300	ZosmaPP2C-03
Cymno03g02260	ZosmaPP2C-13
Cymno03g12120	ZosmaPP2C-06
Cymno04g05930	ZosmaPP2C-12
Cymno04g17020	ZosmaPP2C-08
Cymno04g18000	ZosmaPP2C-11
Cymno04g16690	ZosmaPP2C-14
Cymno05g05290	ZosmaPP2C-09
Cymno05g15240	ZosmaPP2C-14
Cymno05g19410	ZosmaPP2C-11
Cymno11g03910	ZosmaPP2C-01
Cymno14g07340	ZosmaPP2C-10
Cymno04g16690	ZosmaPP2C-18
Cymno04g13900	ZosmaPP2C-19
Cymno05g19410	ZosmaPP2C-17
Cymno05g15240	ZosmaPP2C-18
Cymno05g10160	ZosmaPP2C-16
Cymno01g07920	ZosmaPP2C-21
Cymno04g16690	ZosmaPP2C-23
Cymno05g15240	ZosmaPP2C-23
Cymno05g04150	ZosmaPP2C-24
Cymno05g08590	ZosmaPP2C-22

Collinear PP2C member 1	Collinear PP2C member 2
Cymno06g06460	ZosmaPP2C-21
Cymno09g00710	ZosmaPP2C-25
Cymno11g00480	ZosmaPP2C-21
Cymno11g10760	ZosmaPP2C-25
Cymno14g07340	ZosmaPP2C-26
Cymno14g07340	ZosmaPP2C-26
Cymno02g09720	ZosmaPP2C-33
Cymno02g08640	ZosmaPP2C-31
Cymno03g07820	ZosmaPP2C-31
Cymno06g05150	ZosmaPP2C-28
Cymno07g04950	ZosmaPP2C-29
Cymno08g01570	ZosmaPP2C-27
Cymno06g05730	ZosmaPP2C-42
Cymno06g01520	ZosmaPP2C-40
Cymno13g01500	ZosmaPP2C-41
Cymno16g03120	ZosmaPP2C-36
Cymno16g02870	ZosmaPP2C-39
Cymno01g01030	ZosmaPP2C-50
Cymno01g11360	ZosmaPP2C-46
Cymno01g12760	ZosmaPP2C-45
Cymno02g02660	ZosmaPP2C-48
Cymno03g12120	ZosmaPP2C-48
Cymno03g09860	ZosmaPP2C-47
Cymno11g03910	ZosmaPP2C-44
Cymno12g00930	ZosmaPP2C-45

Between eelgrass and *Posidonia oceanica*

Collinear PP2C member 1	Collinear PP2C member 2
Posoc01g11140	ZosmaPP2C-09
Posoc01g37740	ZosmaPP2C-08
Posoc01g39650	ZosmaPP2C-11
Posoc01g12190	ZosmaPP2C-12
Posoc01g37030	ZosmaPP2C-14
Posoc01g38470	ZosmaPP2C-15
Posoc02g00710	ZosmaPP2C-07
Posoc02g22690	ZosmaPP2C-03
Posoc02g07730	ZosmaPP2C-13
Posoc02g29550	ZosmaPP2C-06
Posoc04g07800	ZosmaPP2C-01
Posoc06g15590	ZosmaPP2C-10
Posoc09g11870	ZosmaPP2C-04
Posoc01g37030	ZosmaPP2C-18
Posoc01g31520	ZosmaPP2C-19
Posoc01g22310	ZosmaPP2C-16
Posoc01g08860	ZosmaPP2C-24
Posoc01g37030	ZosmaPP2C-23
Posoc01g18880	ZosmaPP2C-22
Posoc04g01040	ZosmaPP2C-21
Posoc06g15590	ZosmaPP2C-26
Posoc06g08860	ZosmaPP2C-25
Posoc08g05440	ZosmaPP2C-25
Posoc02g13300	ZosmaPP2C-33
Posoc02g19610	ZosmaPP2C-31
Posoc03g19260	ZosmaPP2C-28
Posoc05g02870	ZosmaPP2C-27
Posoc03g26220	ZosmaPP2C-40
Posoc06g08860	ZosmaPP2C-43
Posoc09g11870	ZosmaPP2C-37
Posoc09g07280	ZosmaPP2C-39
Posoc09g05690	ZosmaPP2C-36
Posoc10g03570	ZosmaPP2C-41
Posoc02g29550	ZosmaPP2C-48
Posoc02g24520	ZosmaPP2C-47
Posoc03g02190	ZosmaPP2C-50
Posoc03g23760	ZosmaPP2C-45
Posoc03g20940	ZosmaPP2C-46
Posoc04g07800	ZosmaPP2C-44

Between eelgrass and *Thalassia testudinum*

Collinear PP2C member 1	Collinear PP2C member 2
Thate01g34000	ZosmaPP2C-11
Thate01g32500	ZosmaPP2C-14
Thate01g32130	ZosmaPP2C-08
Thate04g06570	ZosmaPP2C-01
Thate05g11210	ZosmaPP2C-06
Thate06g00330	ZosmaPP2C-15
Thate06g00860	ZosmaPP2C-11
Thate06g02580	ZosmaPP2C-14
Thate09g13380	ZosmaPP2C-04
Thate01g34000	ZosmaPP2C-17
Thate01g32500	ZosmaPP2C-18
Thate06g00860	ZosmaPP2C-17
Thate06g02580	ZosmaPP2C-18
Thate01g32500	ZosmaPP2C-23
Thate02g26480	ZosmaPP2C-24
Thate03g01980	ZosmaPP2C-21
Thate04g25020	ZosmaPP2C-22
Thate05g02090	ZosmaPP2C-25
Thate06g02580	ZosmaPP2C-23
Thate07g01030	ZosmaPP2C-25
Thate02g35470	ZosmaPP2C-28
Thate03g24890	ZosmaPP2C-29
Thate04g22560	ZosmaPP2C-27
Thate08g01970	ZosmaPP2C-33
Thate09g00400	ZosmaPP2C-27
Thate02g32010	ZosmaPP2C-40
Thate03g16300	ZosmaPP2C-39
Thate06g14500	ZosmaPP2C-40
Thate08g19210	ZosmaPP2C-43
Thate09g07530	ZosmaPP2C-36
Thate09g13380	ZosmaPP2C-37
Thate04g06570	ZosmaPP2C-44
Thate05g11210	ZosmaPP2C-48
Thate06g15750	ZosmaPP2C-45
Thate07g04460	ZosmaPP2C-48
Thate08g15130	ZosmaPP2C-49

Figure S1. GO terms enriched for genes associated with the PP2Cs that show specific high expression in male-flower.

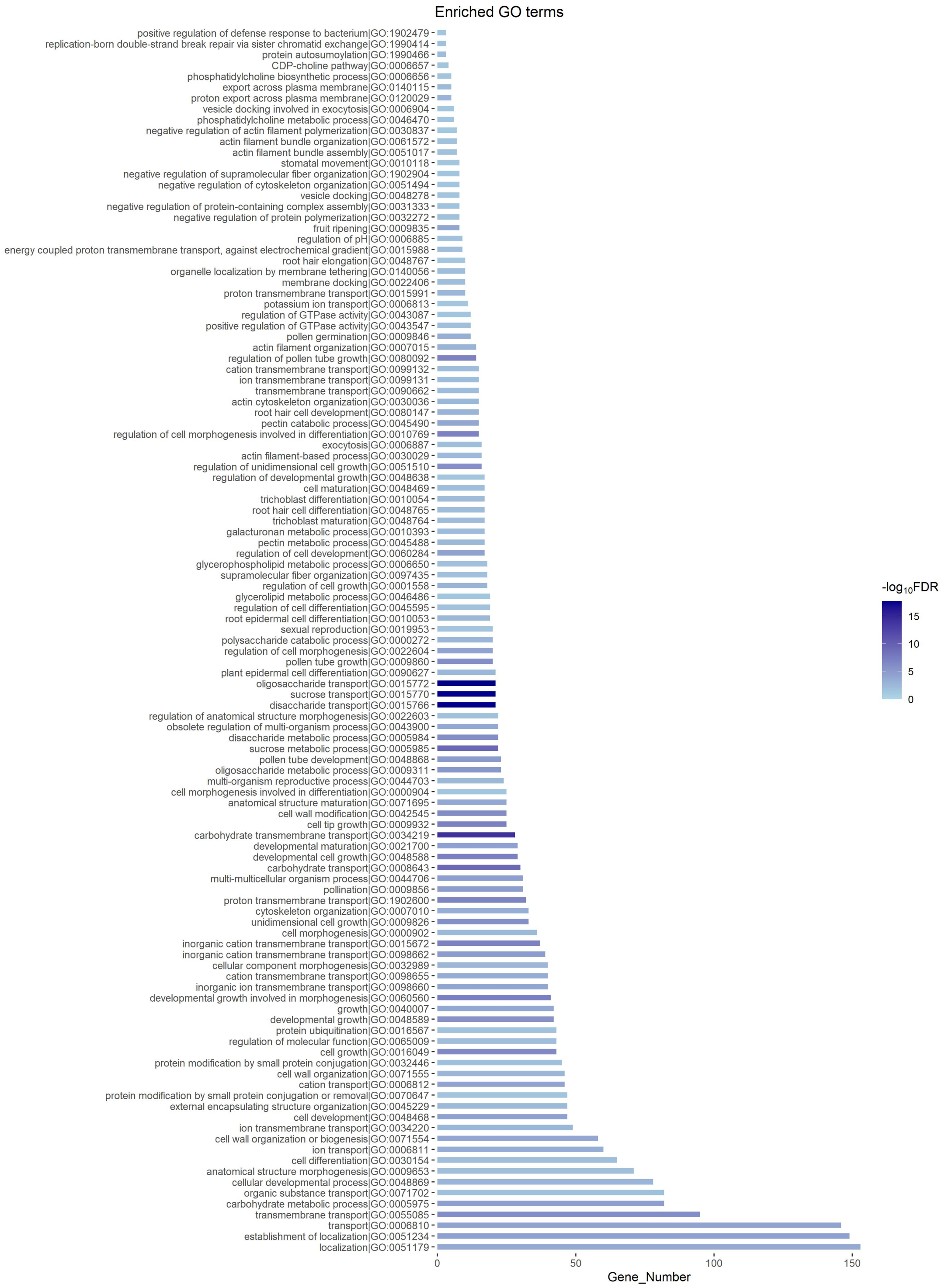

Figure S2. GO terms enriched for genes associated with the PP2Cs that show specific high expression in leaves.

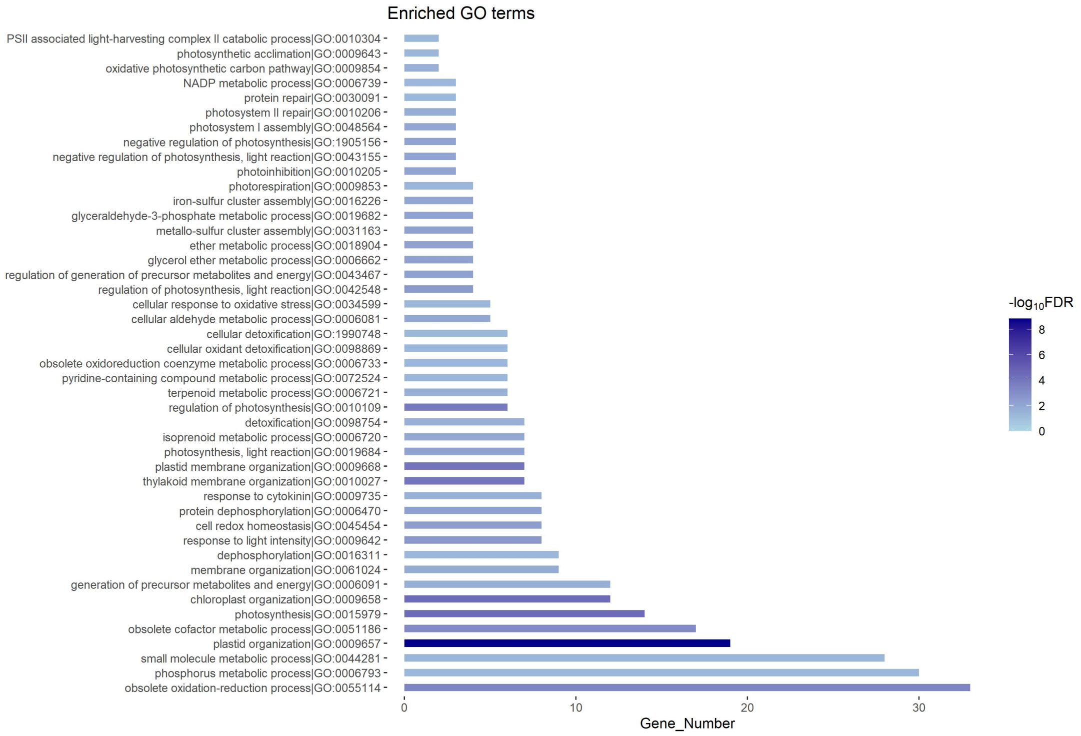

Supplement: Supplementary file 1 [file genes-16-00657-s001.zip › genes-3637947-supplementary.pdf]
